# Effect of body position on peak expiratory flow during mechanical insufflation–exsufflation in people with cervical spinal cord injury: a pilot study

**DOI:** 10.1038/s41598-023-43256-x

**Published:** 2023-10-02

**Authors:** Sung Eun Hyun, Wonjae Hwang, Hye Min Ji, Hyung-Ik Shin

**Affiliations:** 1 Department of Rehabilitation Medicine, Seoul National University College of Medicine, 101 Daehak-ro, Jongno-gu, Seoul, 03080 Republic of Korea; 2 Department of Rehabilitation Medicine, Seoul National University Hospital, Seoul, Republic of Korea; 3 Veterans Medical Research Institute, Veterans Health Service Medical Center, Seoul, Korea

**Keywords:** Health care, Medical research

## Abstract

This prospective pilot study investigated the influence of body position on peak cough flow (PCF) during mechanical insufflation–exsufflation (MI-E) treatment in people with tetraplegia. Fifteen participants with cervical spinal cord injury (C-SCI) were randomized into two groups, which differed in the starting position, that is, the patients were either supine or reclined. Four sessions of MI-E in alternating positions with each session comprising three different maneuvers: five voluntary coughs, five MI-E-assisted coughs, and five MI-E-assisted with manual thrusts were performed with continuous airflow measurement reporting PCF from every cough. PCF was associated with the application maneuvers, total insufflation volume (TIV), and interaction between position and maneuvers but not with the application position. The estimated mean PCF was 1.808, 3.529, and 3.925 L/s when supine and 1.672, 3.598, and 3.909 L/s when reclined from voluntary cough, MI-E, and MI-E with manual thrust, respectively. The estimated PCF change compared to voluntary cough was 1.721 (95% CI, 1.603–1.838) L/s from the combined MI-E and 2.116 (95% CI, 2.005–2.228) L/s from the MI-E with manual thrust, calculated from the linear mixed-model analysis. PCF moderately correlated with TIV (R^2^ = 0.64). Therefore, either position can be used for C-SCI patients as long as MI-E can be performed with manual thrust and sufficient TIV is provided.

## Introduction

Respiratory and abdominal muscle weakness due to cervical spinal cord injury (C-SCI) results in a high mortality burden due to respiratory failure, including atelectasis, pneumonia, and ventilator failure^1^. Additionally, C-SCI results in an impaired ability to cough and breathe deeply, the degree of which depends on the level and severity of the injury^2^. Various pulmonary hygiene techniques are recommended to support adequate ventilation in C-SCI, including postural drainage, assisted cough, tracheal suctioning, and intrapulmonary percussive ventilation.

As one such airway clearance technique, mechanical insufflation–exsufflation (MI-E) is a noninvasive cough aid that augments in/exsufflation volume and flow by inducing a series of positive and negative pressures followed by a short pause. Gradual application of positive pressures to ensure sufficient inhaled volume to mobilize secretion and induce rapid shifts to negative pressures to produce a high expiratory flow can move secretions toward the mouth, allowing them to be easily removed. Tetraplegic individuals with a reduced peak cough flow (PCF) of less than 4.5–6 L/s (270 L/min)^3–5^, which indicates an ineffective cough, are candidates for frequent MI-E use. The application of MI-E to such patients improves PCF and reduces the frequency of pneumonia or hospitalization for people with neuromuscular weakness, including C-SCI^6–8^. In addition, the results of a study investigating the use of MI-E in C-SCI patients indicated that the patients preferred MI-E over trans-tracheal suction^9^.

However, body position affects pulmonary function in people with C-SCI differently from that in healthy people or those with other neuromuscular diseases (NMD)^2^. A higher forced vital capacity (FVC) has been reported in the supine position than in the sitting position in people with C-SCI, which is the opposite of that in a healthy population^10^. The diaphragm generates the main force for inspiration, as the intercostal and abdominal muscles are auxiliary. Inspiratory excursion of the diaphragm is better in the supine position in tetraplegic people, as the intra-abdominal contents are displaced and the muscle fibers become longer at the end of expiration when the muscle is in a more effective point of its length-tension curve^11^. Conversely, expiratory airflow, which is more important in secretion elimination, cannot be further accelerated by increasing muscle effort in the diaphragm. The maximal expiratory airflow is primarily achieved by increasing the operating lung volumes, which is defined as the volume from the end-expiratory lung volume to total lung capacity^12^. In fact, the forced expiratory volume in 1 s (FEV1) and PCF were reported to be greater in the supine than the sitting position only in people with complete tetraplegia^13,14^, and the disparity in pulmonary function becomes negligible when people with both motor complete and incomplete injuries are included in the analysis^15–17^.

MI-E-assisted cough can generate a sufficient insufflation volume more easily than voluntary or manually assisted cough. This is achieved through the application of external positive pressure to compensate for a deficient FVC. Once the maximal insufflation volume is achieved, the individual’s expiratory effort and strength would contribute less to the production of sufficient PCF^12^. However, when using MI-E, the insufflation volume may also differ depending on position, as the bowel contents in people with C-SCI can be affected by gravity. Although there were a few reports about preferring the supine position for a better spirometric evaluation assessed during voluntary respiration or cough in C-SCI patients^2^, no study has been conducted on the effect of posture on expiratory flow generation when using MI-E.

This pilot study aimed to investigate how body position influences PCF during MI-E treatment in individuals with C-SCI. Knowledge regarding the differences in PCF depending on body position could suggest proper positioning for MI-E use in individuals with C-SCI in clinical practice.

## Results

### Clinical characteristics of the study population

**Figure 1:**
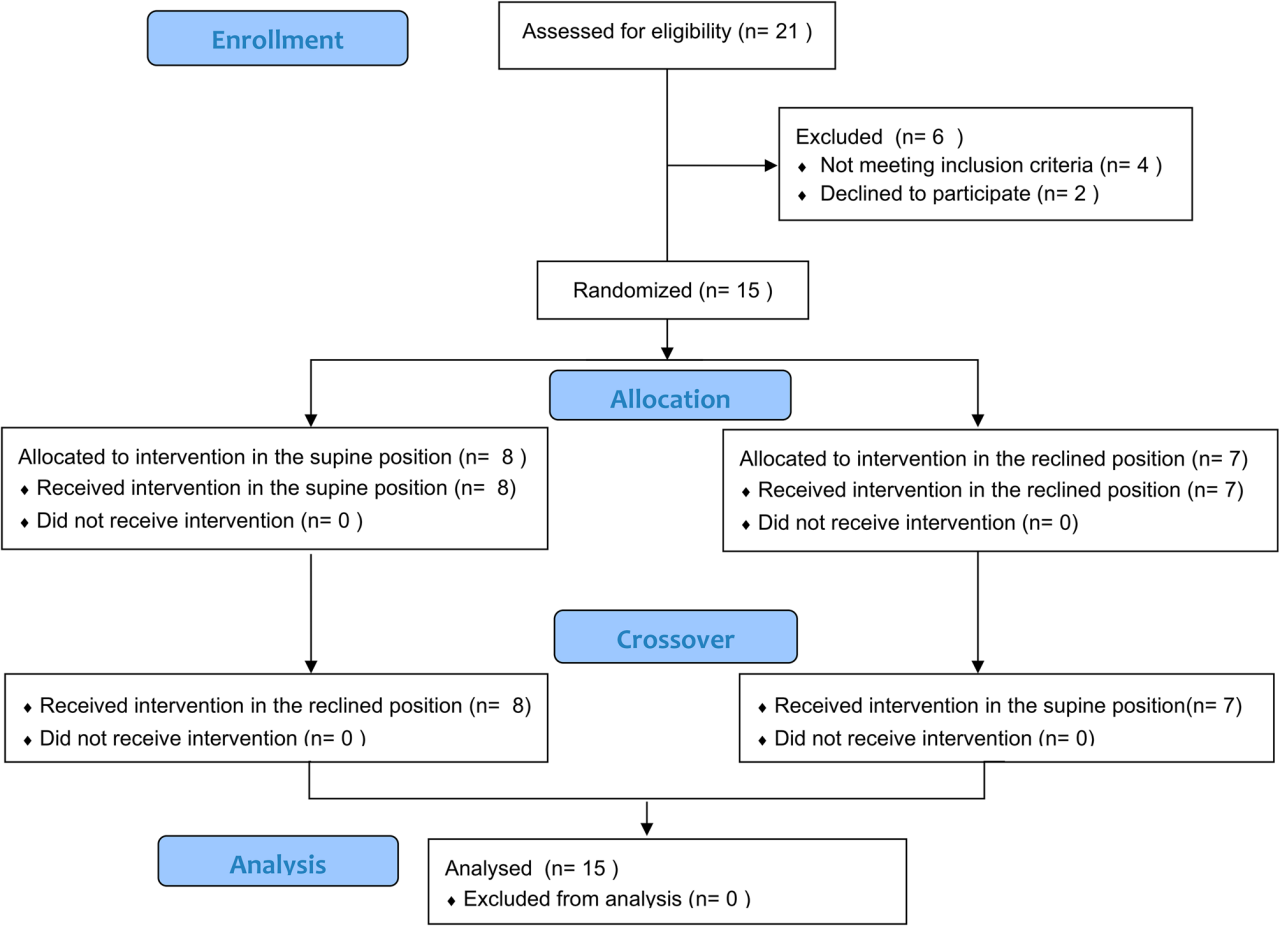
CONSORT flowchart of the study.

Fifteen participants were recruited and randomized into experimental Group I (n = 8) and Group II (n = 7) (Fig. 1). The mean age of the participants was 42.8 ± 16.6 years; the PCF in the supine position was 1.74 ± 0.85 L/s, the level of injury ranged from C3 to C6, and completeness or incompleteness of SCI based on the American Spinal Injury Association (ASIA) Impairment Scale (AIS) was distributed as A in 46.7%, B in 13.3%, C in 33.3%, and D in 6.7%. Table 1 presents the demographic characteristics of the study population. There were no significant differences between the groups in terms of age, onset of SCI, neurological level or severity of SCI, or baseline pulmonary function test (PFT) results. The baseline PFT results demonstrated a position effect only for FVC, maximal inspiratory pressure (MIP), and maximal insufflation capacity (MIC) for all participants, with a preference for the supine position. (Table 2).

**Table 1:** Demographic characteristics of participants.

	Total (n = 15)	Group I (n = 8)	Group II (n = 7)	*p*-value
Gender (M:F)	14:1	8:0	6:1	0.467
Age (years)	42.80 ± 16.60	42.75 ± 17.93	42.86 ± 17.71	0.685
BMI (kg/m^2^)	23.00 ± 2.61	22.36 ± 2.50	23.74 ± 2.92	0.418
Cigarette smoking				
Never	8	4	4	0.530
(pack-years)	27.57 ± 22.10	36.38 ± 25.18	15.83 ± 12.33	
Onset of SCI (month)	9.52 ± 3.11	8.24 ± 3.00	10.97 ± 2.71	0.148
Neurologic level of injury				
C3	1 (6.7%)	0 (0%)	1 (14.3%)	0.490
C4	7 (46.7%)	3 (37.5%)	4 (57.1%)	
C5	4 (26.7%)	3 (37.5%)	1 (14.3%)	
C6	3 (20.0%)	2 (25.0%)	1 (14.3%)	
ASIA classification				
A	7 (46.7%)	3 (37.5%)	4 (57.1%)	0.349
B	2 (13.3%)	2 (25.0%)	0 (0%)	
C	5 (33.3%)	3 (37.5%)	2 (28.6%)	
D	1 (6.7%)	0 (0%)	1 (14.3%)	
Injury mechanism				
Traffic accident	9 (60.0%)	5 (62.5%)	4 (57.1%)	0.726
Fall down	2 (13.3%)	1 (12.5%)	1 (14.3%)	
Slip down	1 (6.7%)	1 (12.5%)	0 (0%)	
Diving	2 (13.3%)	1 (12.5%)	1 (14.3%)	
Others	1 (6.7%)	0 (0%)	1 (14.3%)	
Concurrent illness				
Diabetes	2	2	0	0.400
Hepatitis	1	1	0	
Dyslipidemia	1	0	1	
None	12	6	6	

BMI body-mass index; ASIA American spinal injury association; SCI Spinal cord injury.

Values were expressed as mean ± SD or n (%).

*p*-value was calculated using a Fisher’s exact test for categorical variables and Mann–Whitney for continuous variables.

**Table 2:** Baseline pulmonary function test result from different positions before applying mechanical insufflation–exsufflation sessions.

	Supine	Sitting	*p*-value
PCF (L/min)	134.0 ± 51.9	120.7 ± 55.9	0.123
FVC (L)	2.65 ± 0.74	2.23 ± 0.57	0.011*
FEV1 (L)	2.18 ± 0.73	2.04 ± 0.56	0.125
MEP (cmH_2_O)	36.2 ± 17.7	37.5 ± 19.6	0.683
MIP (cmH_2_O)	71.2 ± 18.9	54.3 ± 13.2	0.002*
MIC (L)	2.74 ± 0.73	2.22 ± 0.58	0.003*

PCF peak cough flow; FVC forced vital capacity; FEV1 Forced expiratory volume in 1 s; MEP maximal expiratory pressure; MIP maximal inspiratory pressure; MIC maximal insufflation capacity.

*p*-value was calculated using Wilcoxon signed-rank test. *p-value < 0.05.

During the MI-E treatment, the highest pre-determined application pressure of +50/−50 cmH_2_O was considered optimal for six participants, +40/−50 cmH_2_O for six participants, +40/−40 cmH_2_O for one participant, and +30/−40 cmH_2_O for two participants. All participants could follow an in/expiratory pressure higher than +30/−30 cmH_2_O, which was provided as the first pressure during the pre-training session. None of the participants complained of chest–abdominal discomfort, hemodynamic instability, or any of the side effects described in the exclusion criteria for discontinuing MI-E treatment during the study.

### Factors associated with PCF

The PCF during voluntary cough significantly differed between the positions with a mean PCF of 1.56 ± 0.51 L/s at supine and 1.36 ± 0.62 L/s at reclined positions, respectively (*p* < 0.001). However, MI-E-assisted cough demonstrated no change in PCF between both applied positions; the reported mean PCF was −3.69 ± 0.48 L/s in the supine and −3.74 ± 0.45 L/s in the reclined position (*p* = 0.09). During the MI-E combined with manual thrust, the mean PCF was even more similar in both positions as 4.05 ± 0.52 L/s in the supine and 4.04 ± 0.57 L/s in the reclined position (*p* = 0.52). The median with interquartile range was 1.56[0.62] L/s, 3.78[0.58] L/s, and 4.19[0.74] L/s in the supine position, and 1.33[0.90] L/s, 3.86[0.50] L/s, and 4.23[0.71] L/s in the reclined position, respectively for voluntary cough, MI-E assisted cough, and MI-E combined with manual thrust. A subgroup analysis, which classified the patients into higher cervical SCI (C3-4, n = 8) and lower cervical SCI (C5-6, n = 7) groups, yielded consistent outcomes. The subgroup analysis demonstrated increasing PCF in the following order: voluntary cough, assistance with MI-E, and MI-E with manual thrust; however, no difference in terms of applied positions was present among both groups.

The linear mixed-model (LMM) analysis demonstrated no main effect of assigned group (*p* = 0.475), treatment day (*p* = 0.094), and session (*p* = 0.381), but only TIV was significant (*p* < 0.001). These results suggested that there was no cumulative effect from consecutive treatment sessions influencing the PCF. Therefore, the final LMM analysis excluded the number of treatment sessions or treatment days as fixed effects and only included the applied position, maneuvers, total insufflation volume (TIV), and the interaction between position and maneuver. The TIV was included because it was significant from the initial analysis.

There was a significant main effect of TIV (*p* < 0.001), maneuver (*p* < 0.001), and interaction between position and maneuver (*p* = 0.001); however, the positions did not show a main effect (*p* = 0.236). The participant-specific random effects were also significant with an estimated intercept of 0.114 (*p* = 0.005) with a residual of 0.118 (*p* < 0.001) (Table 3).

**Table 3:** Linear mixed-effect model summarizing estimated mean differences of peak cough flow values by associated factors.

	est. coeff (SE)	t-value	95% CI
Random effects			
Intercept	0.114(0.006)**		0.056–0.230
Residuals	0.118(0.041)***		0.108–0.129
Fixed effects			
TIV	0.265(0.029)***	9.227	0.209–0.322
Position			
Supine	(reference)		
Recline	−0.136(0.040)	−3.397	−0.215 to −0.058
Maneuver			
Voluntary cough	(reference)		
MI-E	1.721(0.060)***	28.854	1.603–1.838
MI-E with MT	2.116(0.057)***	37.324	2.005–2.228
Interaction			
(MI-E) * (recline)	0.206(0.056)**	3.664	0.096–0.317
(MI-E with MT) * (recline)	0.121(0.057)*	2.143	0.010–0.232

CI, confidence interval; est. coeff, estimated coefficient; MI-E, mechanical insufflation–exsufflation; MT, manual thrust; TIV, total insufflation volume.

*p < 0.05; **p < 0.01; ***p < 0.001.

The final model specification was as follows:

$${\text{PCF}}\sim\beta_{0} + \beta_{1} {\text{Maneuver}} + \beta_{2} {\text{TIV}} + \beta_{3} \left( {\text{Maneuver*Position}} \right) + \gamma \;[{\text{Participant}}] + \varepsilon$$

**Figure 2:**
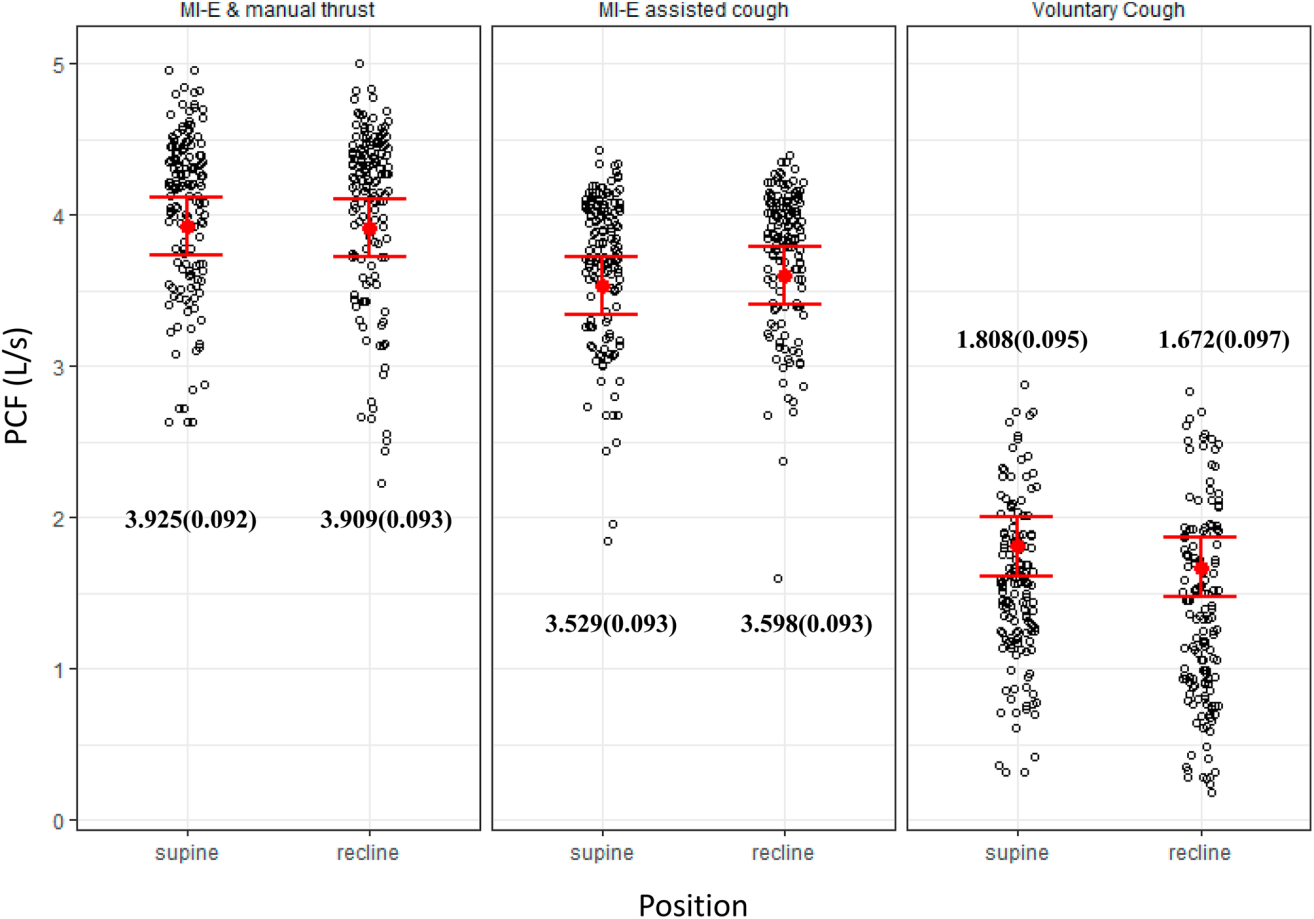
Jitter plots described all measured peak cough flow values with estimated mean and standard error from linear mixed models during voluntary cough, MI-E-assisted cough, and MI-E-assisted with manual thrust, comparing supine and reclined positions, respectively.

After adjusting for TIV and individual differences, the estimated mean PCF [95% confidence interval] was 1.808 [1.610–2.006] L/s in the supine position and 1.672 [1.471–1.873] L/s in the reclined position during voluntary coughing. For MI-E assisted coughs, the PCF increased to 3.529 [3.335–3.723] L/s in the supine position and 3.598 [3.405–3.792] L/s in the reclined position. When MI-E was combined with manual thrust, the PCF further improved to 3.925 [3.732–4.118] L/s in the supine position and 3.909 [3.716–4.103] L/s in the reclined position (Fig. 2). The PCF increased to 1.721 L/s by applying MI-E and 2.116 L/s by additional manual thrust as opposed to voluntary cough. However, this PEF change was affected by the applied position by 0.121–0.206 L/s as the fixed factor of interaction between maneuver and position was significant. A larger TIV also generated a faster PCF with a change of 0.265 L/s, while the PCF change from individual factors (random effects) contributed approximately 0.118 L/s (Table 3).

### Correlation between TIV and PCF

**Figure 3:**
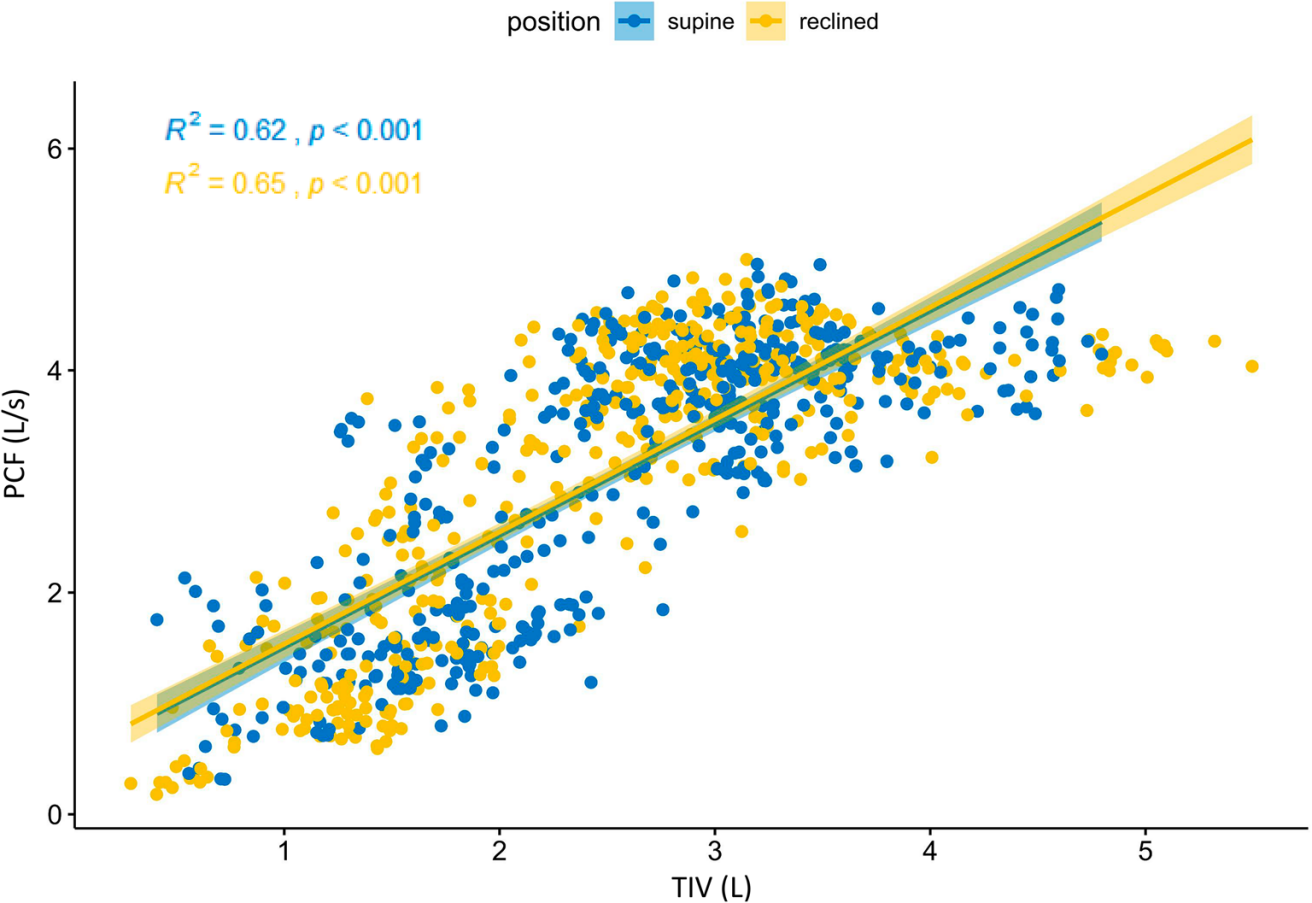
Pearson correlation analysis with confidence intervals between the total insufflation volume (TIV) and peak cough flow(PCF).

Because TIV was significantly associated with the generated PCF during MI-E, the correlation between TIV and PCF was analyzed in terms of the applied position. TIV during voluntary cough showed a difference according to the position (*p* < 0.001), showing a supine position preference with a value of 1.59 ± 0.49 L in the supine position and 1.37 ± 0.43 L in the reclined position. During MI-E assisted cough, its positional difference became negligible as mean TIV was 3.14 ± 0.67 L in the supine position and 3.08 ± 0.78 L in the reclined position (*p* = 0.34), and 3.00 ± 0.73 L in the supine position and 3.04 ± 0.78 L in the reclined position (*p* = 0.26) during MI-E with manual thrust. The correlation between TIV and PCF was moderate^18^ of R^2^ = 0.637 (p < 0.001), demonstrating that a higher TIV was associated with a faster PCF. This correlation effect was also moderate^18^ in each application position (R^2^ = 0.624 in the supine position and R^2^ = 0.648 in the reclined position) (Fig. 3).

## Discussion

Overall, this study revealed that there was no difference in PCF between the supine and reclined positions when MI-E was applied to patients with C-SCI. However, the generated PCF was affected by the application maneuver and MI-E with additional manual thrust resulted in the most substantial increase, approximately 2.116 [2.005–2.228] L/s, compared to voluntary cough. Although the body position itself did not directly alter PCF, the PCF increase from different maneuvers could be influenced by the position, resulting in a larger PCF increase in the reclined position. The provided TIV and individual differences in baseline pulmonary function made a difference of approximately 0.114–0.265 L/s.

The estimated mean PCF [95% confidence interval] was 1.808 [1.610–2.006], 3.529 [3.335–3.723], 3.925 [3.732–4.118] L/s when supine and 1.672 [1.471–1.873], 3.598 [3.405–3.792], 3.909 [3.716–4.103] L/s when reclined from voluntary cough, MI-E, and MI-E with manual thrust, respectively, after adjusting the covariance of TIV and random effects of individual differences. The positional difference of the PCF only existed during voluntary coughing, and the final PCFs generated during MI-E assist or additional manual thrust were not different between the two applied positions. Although voluntary cough did not reach the 2.7 L/s threshold, which is considered essential for efficient sputum clearance from the lungs^3,5^, the MI-E assist or MI-E with manual thrust resulted in PCF values and ranges exceeding 2.7 L/s in both applied positions. However, all measured PCF values fell below the 4.5 L/s cutoff, indicating the necessity for assisted cough^3,5^.

Although voluntary PCF was faster in the supine position compared to the reclined position, the increase in PCF from MIE-assist or additional manual thrust was more pronounced in the reclined position, ultimately resulting in a similar final PCF in both positions. It is also well correlated with the significant interaction factor between maneuver and position (Table 3), indicating the reclined position with MI-E influenced PCF change of approximately 0.206 L/s, and with MI-E combined with manual thrust made additional PCF change of approximately 0.121 L/s. MI-E-assisted cough can easily achieve a higher insufflation volume than normal inspiratory capacity from voluntary maximal breathing in individuals with C-SCI because of their weak respiratory muscles followed by low lung and chest wall excursions. As such, MI-E-assisted cough could generate sufficient PCF as long as sufficient maximal insufflation volume is guaranteed, regardless of body position. Figure 3 suggests that a minimum of approximately 2.5 L of TIV is necessary to achieve a PCF of 2.7 L/s^4,5^.

A previous study investigating 36 patients with NMD suggested that the best individual PCF could be achieved with intermittent positive pressure breathing-assisted insufflation^19^. Herein, the authors argued that even submaximal optimum insufflation capacity (OIC), which was smaller than MIC, from smaller insufflation pressure than the pressure generating MIC can reach the fastest PCF. From the pressure–volume-flow relationship, 30–40 mbar (30.6–40.8 cmH_2_O) generated OIC was suggested to be the candidate for those with NMD to achieve the best PCF. Our study selected the most convenient in/expiratory pressure setting for each participant from a pre-training session, which generated the best PCF from gradually increasing pressure. The most commonly selected pressure settings were +40–50/−50 cmH_2_O, which was relatively higher than that reported in a previous study^19^, indicating that MIC or at least submaximal OIC was sufficiently achieved in both positions. The optimal pressure was selected in the supine position as to be higher than the pressure set in the reclined position, because supine position was suitable for generating higher MIP and FVC in C-SCI patients than in the reclined position. This suggests that the patient’s intra-abdominal contents could be well-displaced during insufflation by MI-E, resulting in the best PCF from both positions.

Previous reports have shown an additional benefit of combining manual thrust with MI-E in patients with NMD, including myopathy or motor neuron disease, however, there is very little information regarding MI-E with manual thrust in tetraplegic individuals. Kim et al.^20^, also measured MI-E-assisted cough using a serially connecting flowmeter and reported that MI-E combined with manual thrust showed a synergistic effect to improve PCF only in the reclined position. Compared to MI-E alone in 40 NMD patients with a mean baseline MEP of 25.3 cmH_2_O, MIP of 19.5 cmH_2_O, and FVC of 0.67 L, whose inspiratory volume and pressure, that is, lung compliance, were severely decreased compared to our participants, MI-E with manual thrust using ± 40 cmH2O pressure generated a PCF of 3.37 L/s, which was better than the value of 2.95 L/s without manual thrust^20^. Patients with NMD with an impaired VC lower than 40% or baseline PCF of < 3 L/s also showed improved PCF through a combination of MI-E and manual thrust compared to either MI-E alone or manually assisted cough^21,22^. The patient satisfaction scale was also reported to be the same between MI-E alone and manual thrust combined with MI-E. Our study investigated C-SCI rather than NMD and reported the same additional benefits of approximately 0.4 L/s from a combination of manual thrust on MI-E. Although the insufflation volume may be sufficient with MI-E alone, further expiratory pressure or force from manual thrust is helpful to generate an even faster PCF.

Although PCF variations caused by the assistance of MI-E or manual thrust have been documented in different populations as mentioned above^19–22^, there is a lack of reports addressing the potential differences in body positioning during MI-E or manual thrust in people with C-SCI. Limited research has provided information about the specific positions used so far. Positional changes in pulmonary function were distinct in tetraplegic patients, as indicated by the results of PFT^2^. While individuals with NMD typically experience uniform muscle weakening, it is important to highlight that C-SCI patients without ventilator support exhibit a disproportionate diaphragm weakness that is relatively less pronounced compared to the almost complete inactivity of intercostal and abdominal muscles^10^. The removal of abdominal organ compression in the sitting position pulls the diaphragm downward and makes it flat, which increases the diaphragm’s demand and effort for breathing^11,12^. Therefore, the outcomes of the current study should be interpreted within the context of C-SCI patients and cannot be generalized to all individuals with NMD.

This study had some limitations. First, we only measured PCF during cough but could not measure the exact amount of eliminated sputum to assess the effectiveness of the procedure in maintaining pulmonary hygiene. Additionally, comparing MI-E-assisted cough at 60° or 90° sitting position can provide more information about the best position for MI-E in people with C-SCI as well. Furthermore, information on long-term pulmonary prognosis, which is affected by the body position applied for MI-E treatment, will further empower future clinical decisions. Last, this pilot study is limited by a small, heterogeneous sample of individuals with varying SCI severities, restricting the generalizability of findings. The estimated sample size for paired t-tests is notably high due to the small mean differences relative to the standard deviations of differences. For instance, n = 557 would be needed for MI-E assisted cough, and n = 80,602 for assessing positional differences during MI-E combined with manual thrust. Therefore, it is imperative to utilize a small sample size but incorporate multiple, repetitive measures using LMM.

In conclusion, the PCF during MI-E does not change according to the position, but a beneficial effect of applying MI-E and additional manual thrust could be achieved in both the supine and reclined positions. Generated TIV during cough was important in determining PCF in both positions. Hence, either position can be used as long as MI-E can be performed together with manual thrust and sufficient TIV is provided.

## Methods

### Study Participants

This prospective, randomized crossover pilot study recruited C-SCI patients with impaired cough who required further cough augmentation, defined by a PCF < 270 L/min^5^. Participants were excluded if they were tracheostomized or required continuous noninvasive ventilation and oxygen support; had any previous restrictive or interstitial lung disease; had any other contraindications for MI-E use, such as pneumothorax, pneumomediastinum, emphysema, or lung barotrauma within 1 month; had any active communicable respiratory infections; were hemodynamically unstable, i.e., systolic blood pressure < 90 or > 160 mmHg, diastolic blood pressure < 50 or > 110 mmHg, respiratory rate > 33/min, heart rate > 130 bpm, or saturation (SpO2) < 95%^7,23^. All participants provided informed consent to participate in this study; then, they underwent PFT in both the supine and sitting positions to evaluate baseline PCF, FVC/FEV1, MEP/MIP, and MIC following instructions of the American Thoracic Society guidelines^24^ before starting the MI-E treatment sessions. This study was approved and monitored by the Institutional Review Board of the Ethical Committee, in accordance with the Declaration of Helsinki (IRB No. NTRH-20018).

### Study design

Eligible participants were randomized in a 1:1 ratio into groups I or II without stratification using a computer-generated block size of 4. Group I started in the supine position, was later reclined to 40° on the first day, and then the reclined position first followed by the supine position on the next day. Conversely, Group II underwent four sessions of MI-E in the opposite order of the applying position. The angle of 40°-reclining was chosen because previous studies have shown that this angle can achieve a more effective manual thrust combined with MI-E than in a 90°-sitting position^20,25,26^.

The study design provides alternating starting positions of either supine or reclined for each day and each group because the cumulative treatment effect can also affect the generated PCF during MI-E. Consecutive MI-E application with eliminated sputum between coughs affects airway resistance and generates better PCF. However, repetitive cough combined with MI-E assistance would result in fatigue because of repeated maximal effort during exsufflation and decreased PCF. Conversely, if more MI-E treatment sessions are completed, more experienced participants can cooperate better with MI-E, which generates a faster PCF. This bias from repetitions of the MI-E application could be avoided by changing the order of the applied posture for every session for all participants.

### MI-E application

MI-E treatment sessions were administered using the CoughAssist E70 (Phillips Respironics, Murrysville, Pennsylvania), which was set to generate 3 s of insufflation, 2 s of exsufflation, and 2 s of rest for one cough, with a low inhale flow setting and oscillation off to simulate physiological coughing and facilitate better cooperation for higher in-exsufflation pressure^27^. A pre-training session was performed in the supine position to determine the personalized pressure setting for each participant. The supine position was chosen for setting the personalized, optimal pressure because a higher FVC was reported in the supine position^10^. This indicates that a higher insufflation pressure is anticipated to generate larger TIV in the supine position compared to the reclined position. The maximal pressure optimized only in the sitting position might be insufficient to generate enough TIV which can reach MIC in the supine position. By gradually increasing the pressure from 30 to 50 cmH_2_O in 5 cmH_2_O increments, the highest pressure that each participant could withstand was measured. If the maximal pressure of ± 50 cmH_2_O was tolerable, then the pressure at which the fastest PCF could be generated was determined as the applied pressure.

After determining the optimal MI-E pressure settings, the protocol comprised five consecutive voluntary coughs following 3 s of full inspiration, 2 s of maximal expiration, and rest was initiated. Participants were instructed to cough five times into the facemask while wearing a well-sealed mask (Air Way 96 mm (#4), ACE Medical Co., South Korea) with support from a physiotherapist or caregiver to prevent any air leak. After eliminating the expelled sputum, one set of five MI-E-assisted coughs was provided, followed by another set of five more MI-E-assisted coughs with manual thrusts. The three sets (5 coughs/1 set) of different application maneuvers—voluntary cough, MI-E assist, and MI-E with manual thrust—comprised one session at each position, and these three sets were repeated in the same order at the next assigned position.

When performing MI-E combined with manual thrust, the physiotherapist asked the participant to cough at the start of the exsufflation period while delivering manual pressure to the participant’s bilateral costophrenic angle toward the direction of the upper chest simultaneously. An experienced physiotherapist blinded to the analysis of the mean PCF conducted all MI-E treatments and measured the concomitant airflow. A separate researcher blinded to the assigned group and applied position performed all the analyses.

### Expiratory flow measurement

The in-expiratory airflow generated in both directions during the MI-E session was measured using a flowmeter (CITREX H4 gas flow analyzer, IMT Analytics, Buchs, Switzerland) serially connected to one side of a single-use antibacterial filter towards the facemask interface, and the other side to the CoughAssist E70. This flowmeter was formally calibrated and validated annually by IMT Analytics to ensure that the maximal uncertainty error was within 0.75% for all measurements. Expiratory airflow was measured as a negative value; therefore, the lowest value during exsufflation was the PCF, which could be calculated from each cough with continuous airflow measurement^28^. The total insufflation volume (TIV) was calculated through integration by squaring insufflation time and flow, as follows: volume (L) = time (s) × flow (L/s).

### Statistical analysis

The LMM was used to estimate mean PCF, standard error, and confidence interval which described the changes in the PCF according to the position or maneuver. Applied position, maneuvers, TIV, and interaction between maneuver and position were included as fixed effects, and participant-specific random intercepts were included as a random effect to account for repeated measures within participants covering individual, different baseline pulmonary functions. The normality test was confirmed for residuals. The strength of the linear correlation between PCF and TIV was analyzed using Pearson’s correlation analysis after calculating the correlation coefficient R^2^ overall and in each application position. Demographic data comparison between the assigned groups was performed using the Mann–Whitney or Fisher's exact test. Baseline PFT outcomes and measured PCF values were compared between the two applied positions using the Wilcoxon signed-rank test after normative analysis. All analyses were performed using the R software version 4.2.2.

### Ethics declaration

This study was approved and monitored by the Institutional Review Board of the Ethical Committee, in accordance with the Declaration of Helsinki (IRB No. NTRH-20018).

## Data Availability

The data generated and/or analyzed during the current study are available from the corresponding author upon reasonable request.
